# Supplementary material for: Acceptance and compliance with micronutrient powder (MNP) among children aged 6–23 months in northern Nigeria
Source: PLOS Glob Public Health. 2022 Oct 17;2(10):e0000961. doi: 10.1371/journal.pgph.0000961 (PMC10022258; doi:10.1371/journal.pgph.0000961)
Supplement: S1 Dataset — (ZIP) [file pgph.0000961.s008.zip › S8_Dataset/In-depth Interviews.zip!/In-depth Interviews/030715_K_BK_AB_2_CG.c.pdf]

I: Good morning Ma, I want you to describe for me the people in your immediate family living in your household

R: We are actually many

I: What about children from six months to twenty three months

R: They are four

I: How many are boys and how many are girls?

R: Three girl's one boy

I: Thank you very much. We have heard of food challenges for young children in this setting related to the seasons, like in this raining season what are the types food that are difficult to get

R: We have all the foods, we don't lack any particular type of food.

I: Some caregivers said they give their children tea, what is tea?

R: (Laughing) I always see people buying to give their children, I don't know what it is made up off although like biscuits and milk can be added into it

I: Last time you were given MNP to give to your child, could you tell me about your experiences with the product?

R: I don't have any problem with it.

I: When you started administering the MNP to him, can you share with me your experiences

R: When I finished preparing kunu, I divided it into three, one of the portions I open the MNP and pour it inside the kunu stir it and I gave it to her and she drank it all. That was how I continue giving her. But these days she refused taking it and I had to use force feeding on her.

I: You she does not take kunu anymore

R: Yes except tuwo

I: Why is it that she does not take kunu any longer

R: It is because she always have food all the time to eat off to the extent she has abandoned kunu. But if I use the MNP with kunu she does take it.

I: You mean she always take the one that contain the MNP

R: Yes, she takes the one that contain the MNP but after that one she will not take drink anymore

I: Since you started given him the product, what could say about his body?

R: Like how

I: Like increasing her eating habit

R: Yes, it has increase her eating habit

I: What could you say about her health since you started giving her the MNP

R: She has been normal, no headache, vomiting or stooling except yesterday her body temperature increases with some rashes but now she is okay. And again anything she is eating she eat to her satisfaction

I: There are some women that said it was hard to store the MNP because some children will come and steal it, some eaten by rodents. Tell me about your experience

R: The first time, it was the man that brought it and open it after then, anytime I open it and use it I always kept it properly. There is nothing like rodents or even children that have ever tempered with it.

I: Since you started giving her the MNP is there any challenge or problem occur as a result of using the MNP?

R: No, there is no challenge or problem faced since I started using it

I: Some caregivers said they do forget to give their child the MNP, do you have similar problem?

R: As for me I always give her in the morning immediately I finish sweeping and bathing, I quickly make her kunu and use the MNP for her

I: What always make you to remember all the time and if you are to give advice to those caregivers who always forget

R: Since I know I have been giving a task on her to be giving this MNP to her in the morning and not in the afternoon, I am always conscious of it. When I woke up in the morning washed her face and both her then administer the MNP to her

I: Like if you have a friend who always forget, what kind of advice would you give her

R: For example if you go for an occasion and forget to give your child, instead of 30 days you will use 31 days for a sachet and if you missed two days it therefore means that you will use 32 days to complete the sachet. Like me I have not missed a single day without giving her the MNP

I: You said you always give her MNP mix with kunu which type of kunu

R: It is koko made of millet either hot or cold one depends on which one is available.

I: Like the first you started giving the MNP did she noticed any different taste of koko

R: Yes she noticed the taste is different because when I first gave, she squeeze her mouth wanting not to take at the end I had to use force feeding on her

I: Do you think she like this MNP?

R: She doesn't like it I always force her

I: You said since she started taking the MNP it has increase her food intake (cut in)

R: Yes it has increase her food intake and again she has not falling sick again

I: Which type of food does she eat now that has increase?

R: Tuwo, kosai or even biscuits

I: What about koko

R: The rate at which she takes koko has reduced but she eats a lot of tuwo in such a way that she hardly takes breast milk

I: What about the rate at which she takes breast milk

R: She takes breast milk a lot at times I will use force to remove her mouth from my breast

I: Thank you very much. In other communities we notice that the sharing of the MNP occurred, could you talk about any sharing of the MNP when you used it with another woman

R: I have not given it to anybody. Because since the very day I collected her father said it is mainly for her I now went and kept

I: Is there any day that she drank the MNP and remains?

R: She hardly finished it at times

I: What happen to the remaining?

R: The remaining I keep and add some water, later when she is hungry I will give her

I: You know were given 60 sachets for 60 days do you think it will be enough for 60 days?

R: It will not be enough and it is good for two sachets to be given each day

I: Thank you very much. Now that you have used the MNP, could you tell me how you perceive it?

R: I considered it to be a medicine

I: Why did you considered it to be a medicine

R: Because I was told to be giving one each day and that is how I normally give them medicine

I: Now that you have used it, would you purchase it if it were cheaply on sale in this community?

R: I don't really know how much it can be sold

I: How much do you think you can but it supposing it is taken to the market?

R: It should not be more than fifty naira (N50)

I: Thank you very much for your reach explanations. Now tell me some ways that you believe would be effective for promoting MNP to caregivers in this community

R: This question actually I don't know

I: I mean if this product is brought to the community, whom do you think that can give the information about the product?

R: You Know anything that is brought into the community most first all be taken to the village head, it is there he will know what to do

I: In this community who are the people that community members respect and obey their instruction

R: It is the village head advice

I: And who again

R: They are the only people in this community. They are the only people when they said to the people do this, they will do it

I: Like in breast feeding the babies who normally gives you advice especially when you just put to bed

R: Is my mother that first thought me

I: Like now if this MNP is brought to the community, where do like to hear more information about it

R: Frankly speaking, I don't were that person should go and get the information or the other way round the person can go to the district head for further information?

I: Apart from the father of the child and the district head, what form of media do you prefer for receiving MNP messages?

R: Poster can be use and radio

I: Can tell me what made the implementation of tamowa to be successful that you will like us to follow with this MNP

R: The people that first used it were cures of tamowa and it was not first brought here. They stand in the cue and they were giving in an orderly manner

I: If you are ask to talk to another female caregiver about this MNP what will you tell her

R: I will tell to make kunu and divide it into three places and put one sachet of the product and stir it in a cup then give it to him/her to drink.

I: Then how will you tell her not share it with any child or anybody

R: I will tell her not share it with anybody he is the only person only should be given. And she should find a good/safe place and keep it and after she finished using each sachet she should keep the empty sachet so that even if they come to check they will see it for recording purposes

I: Now we have come to the end of this interview I don't know if you have any problem, question about the product since the very day you started using it on your daughter

R: The sachet at times is somehow difficult to open

I: Do you have any advice to give

R: My advice is that any one that will not open easily should be use by scissors or razor

I: Thank you very much malama Maryam.
